# Novel Rhinological Application of Polylactic Acid—An In Vitro Study

**DOI:** 10.3390/polym15112521

**Published:** 2023-05-30

**Authors:** M. P. Gowrav, K. G. Siree, T. M. Amulya, M. B. Bharathi, Mohammed Ghazwani, Ali Alamri, Abdulatef Y. Alalkami, T. M. Pramod Kumar, Mohammed Muqtader Ahmed, Mohamed Rahamathulla

**Affiliations:** 1 Department of Pharmaceutics, JSS College of Pharmacy, JSS Academy of Higher Education and Research, Mysuru 570015, Karnataka, India; 2 Department of ENT, JSS Medical College and Hospital, JSS Academy of Higher Education & Research, Mysuru 570004, Karnataka, India; 3 Department of Pharmaceutics, College of Pharmacy, King Khalid University, Abha 61421, Saudi Arabia; 4 Department of Pharmacy, Mental Health Hospital, Ministry of Health, Abha 61421, Saudi Arabia; 5 Department of Pharmaceutics, College of Pharmacy, Prince Sattam Bin Abdul Aziz University, Al Kharj 11942, Saudi Arabia

**Keywords:** sinusitis, nasal stents, 3D printed nasal stents, endoscopic sinus surgery, biomaterials, biomaterials in 3D printing

## Abstract

A novel approach to the treatment of sinusitis is the use of nasal stents. The stent is loaded with a corticosteroid, which prevents complications in the wound-healing process. The design is such that it will prevent the sinus from closing again. The stent is 3D printed using a fused deposition modeling printer, which enhances the customization. The polymer utilized for the purpose of 3D printing is polylactic acid (PLA). The compatibility between the drugs and polymers is confirmed by FT-IR and DSC. The drug is loaded onto the polymer by soaking the stent in the drug’s solvent, known as the solvent casting method. Using this method, approximately 68% of drug loading is found to be achieved onto the PLA filaments, and a total of 72.8% of drug loading is obtained in terms of the 3D-printed stent. Drug loading is confirmed by the morphological characteristics of the stent by SEM, where the loaded drug is clearly visible as white specks on the surface of the stent. Drug release characterization is conducted by dissolution studies, which also confirm drug loading. The dissolution studies show that the release of drugs from the stent is constant and not erratic. Biodegradation studies were conducted after increasing the rate of degradation of PLA by soaking it in PBS for a predetermined duration of time. The mechanical properties of the stent, such as stress factor and maximum displacement, are discussed. The stent has a hairpin-like mechanism for opening inside the nasal cavity.

## 1. Introduction

**Figure 1:**
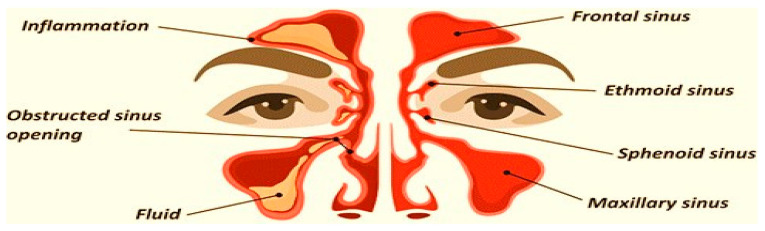
Normal sinus versus inflamed sinus, blocking fluid drainage.

**Figure 2:**
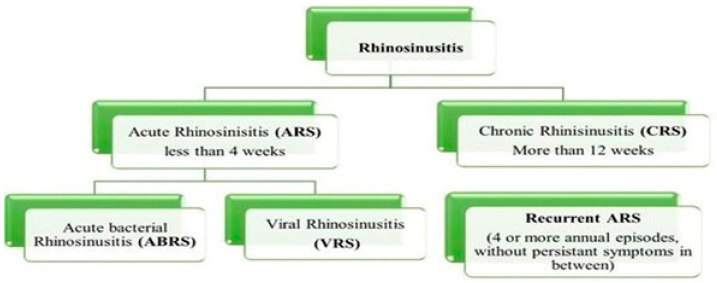
Classification of rhinosinusitis based on duration of prevalence.

Sinusitis is a very common condition affecting the nasal mucosa and paranasal sinus (PNS). It is a condition in which the air-filled sinuses become chronically inflamed, obstructing natural fluid drainage and causing unbearable facial pain [1,2]. The preferred term is rhinosinusitis, as sinusitis most often goes together with inflammation of the contiguous nasal mucosa. The normal sinus versus inflamed sinus, blocking fluid drainage, and classification of rhinosinusitis are represented in the schematic diagram, in Figure 1 and Figure 2, respectively. Approximately 15% of the Indian population suffers from this condition. Endoscopic sinus surgery (ESS) is a surgical approach to this condition where the inflamed, diseased tissue is surgically removed. Post-surgical treatments include douching, various topical and oral anti-inflammatory drugs, and antibiotics [3,4].

Some of the currently used treatment strategies and pharmaceutical dosage forms for the treatment of rhinosinusitis are compiled in Table 1 [5]. Currently, available dosage forms are immediate-release, and they are not effective in preventing scar tissue formation, polyp growth, and inflammation due to the surgical wound; all of which result in the sinuses closing up again, requiring secondary surgical and other interventions [6,7]. This is both a risk to patient compliance and not economically feasible. Several novel strategies have been put forward to make sustained-release dosage forms as well as precautions to prevent the need for secondary surgical interventions. Nasal stents are an innovative and novel approach to the management of rhinosinusitis. Stents are devices that temporarily keep the cavity open, promote the healing of wounds, and alleviate blockage; however, the FDA considers any device that can be placed into a surgically or naturally formed cavity for 30 days or more to be a stent. Nasal dilators are devices that mechanically open your nasal passages, reducing the resistance to incoming air without the use of medication [8,9,10]. The descriptions about maxillary sinus stenting, and middle turbinate stenting are also available; Propel™ stent, Sinu-Foam™ spacer, MicroFlow spacer and Relieva stratus™ are some of the currently available and clinically approved stents [11,12,13,14].

PROPEL^TM^ is a biodegradable nasal stent loaded with a corticosteroid, mometasone furoate. It is designed to slowly release the drug in a sustained manner over a period of 30 days, disintegrating in the process. It also has one more function, which is preventing the closure of sinuses again after surgery. Considering all these facts in the present research, the stent is designed in such a way that it adheres to the walls of the ethmoid sinus and prevents it from closing again due to inflammation or scar tissue formation. PROPEL^TM^ is developed by intersect ENT, which is made of a polylactide-co-glycolide polymer matrix, which encloses a dose of 370 µg of the corticosteroid, which is mometasone furoate.

Mometasone furoate (MF) was used as a drug because it acts as a corticosteroid, which treats nasal congestion, allergic rhinitis, asthma, nasal polyps, dermatitis and pruritis. MF acts by inhibiting mast cells, eosinophils, basophils, and lymphocytes. It binds to the glucocorticoid receptor which leads to a series of events that enhance the expression of molecules that fight inflammation or decrease the expression of molecules that cause inflammation (such as interleukins 4 and 5). MF reduces inflammation by blocking transcription factors such as activator-protein-1 and nuclear factor kappa B (NF-kappaB). The polylactide–co–glycolide polymer is used in the development of stents as it has good biodegradation properties and is highly compatible with mometasone furoate.

The development of biodegradable nasal stents is an area of active research in the field of biomedical engineering. These stents are used to treat various nasal conditions, such as chronic sinusitis and nasal valve collapse, by providing support to the nasal passage while it heals. Traditional stents made of silicone or other non-biodegradable materials can cause tissue damage, inflammation, and infection. Biodegradable stents are designed to eliminate these risks by gradually degrading in the body and eliminating the need for removal procedures. The materials used in these stents are carefully selected to ensure that they degrade safely and do not cause any adverse effects on the body. Overall, the development of biodegradable nasal stents has the potential to greatly improve patient outcomes and reduce the need for repeated procedures.

The nasal stent is intended to deliver the drug in a controlled, localized, and direct manner to the mucosal tissue. The physician assists in stenting the device, post-ESS. Its spring-like design props open when inserted through a silicone catheter. The spacing function provided by the stent along with the loaded drug, not only assists in wound healing and decreases inflammation, but also minimizes scar tissue and granulation tissue formation over a period of 30 days before its complete disintegration. In the current research study, novel nasal stents are prepared by the 3D printing method. The designed stent was subjected to drug-loading and then evaluated for various parameters such as mechanical properties, biodegradation studies, and drug release.

## 2. Materials and Methods

### 2.1. Materials

The Active Pharmaceutical Ingredient (API) used for the purpose of this study was Mometasone Furoate (MF), which was obtained from Coral Drugs Pvt Ltd., Sonepat, Haryana, India. The polymer of choice was Polylactic Acid (PLA) from Imaginarium Solutions (I) Pvt Ltd. Andheri (east) Mumbai, Maharashtra, India. Thermoplastic Polyurethane (TPU) was obtained from Shenzhen SUN Industrial Co., Shenzhen, China. The solvents utilized were ethanol, acetone and, chloroform, all were obtained from Loba Chemie Pvt. Ltd., Mumbai, Maharashtra (India).

### 2.2. Methods [15,16,17,18]

#### 2.2.1. Preformulation Studies API Characterization of Mometasone Furoate (IP)

The characterization of the API was conducted in terms of appearance, solubility, UV analysis, and heavy metal concentration. The analysis report is provided by Coral Drugs Private Limited Co; from where the drug was procured. Another criterion was the determination of absorption maxima of Mometasone furoate (λmax) where spectrum scanning of the drug was conducted with an Ultra Violet (UV) Vis spectrophotometer in the range of 200–400 nm.

#### 2.2.2. Specification Details of the Polymer (PLA)

The 3D printing substance (PLA) is a biodegradable, hard, robust material obtained from plant starch instead of crude oil. PLA prioritizes appearance and strength over toughness. PLA is a biodegradable thermoplastic derived from renewable sources, such as sugar sticks, even potato starch or tapioca roots, cornstarch,

Specifications:

- It is naturally transparent and can be colored to varying degrees of translucency;
- It is strong and more rigid than other materials used in 3D printing;
- It warps and shrinks less than other materials;
- Generally, printed objects will have a glossier look and feel;
- Flexural Strength: 88.8 MPa, Tensile Strength is 61.5 MPa, Melting Temperature: >155 °C, Heat Resistance: 110 °C, and Impact Strength: 30.8 [kJ/m^2^],
- Elongation at Break: 6%, Minimum wall thickness: 0.0197 mm to 0.5 mm; Extruded temperature: 160–220 °C; Shore hardness:85 A; PLA density: 1.25 g/cm^3^; Standard tolerance: ±0.05 mm and the thermal conductivity is 0.13 W/m-K

#### 2.2.3. Characterization of Optimized Formulation

##### Fourier Transformed Infrared (FT-IR) Spectroscopic Analysis

The FT-IR analysis was employed to check for interactions among drugs and polymers; if any. The process of sample preparation involved triturating the drug and polymer with the help of a mortar and pestle. In KBr powder, the mixture of polymer and drug is dispersed in a ratio of 1:4. This mixture was pressed into a pellet using a KBr press at a pressure of 600 kg/cm^2^. After placing this sample in the sample cell, a spectral measurement was taken. Between the regions of 4000 cm^−1^ and 400 cm^−1^; the wavenumber was obtained by the diffusion of spectra powder reflectance in an FT-IR spectrophotometer instrument (Shimadzu FT-IR 8400S, Kyoto, Japan).

##### Scanning Electron Microscopy (SEM) Analysis

A Scanning Electron Microscope (JEOL JSM-IT300 InTouchScope™, Tokyo, Japan) was used to study the surface morphology and topography; as well as to confirm the loading of the drug onto the stent. With the help of double-sided tape, the stent was firmly attached to the aluminum stub. A thin layer of gold was sputter-coated onto this arrangement, which induced conductivity in the specimens. After processing the sample, it was then subjected to SEM analysis.

##### Differential Scanning Calorimetry (DSC)

The DSC technique was also utilized along with FT-IR to check the compatibility between the polymer and the drug used. A differential scanning calorimeter (Shimadzu DSC-60, Kyoto, Japan) was used to conduct this technique. DSC thermograms were obtained for the pure drug and the final drug-loaded stent. The sample was prepared by filing the drug-loaded stent at different locations with the help of a nail file. Alpha-alumina discs; made into empty cells of high purity served as the reference for measurements of calorimetry. The sample was heated at the rate of 10 °C/min in an inert atmosphere purged with nitrogen (N_2_), and the energy in J/Kcal was measured.

#### 2.2.4. 3D Printing of the Finalized Design

Once the design was finalized, the .STL file format, which is compliant with 3D printing, was created with the help of a professional. Flash forge finder 2.0 3D Printer (Zhejiang Flashforge3D Technology Co., LTD, Jinhua, Zhejiang, China), which is a Fused Deposition Modelling (FDM) type of 3D Printer was utilized for the purpose of this study. The problem regarding 3D Printing, which arose due to the inconsistencies in the .STL file design was mitigated by virtually slicing the entire design, layer by layer to find the problem area; mitigate it and proceed with printing [19].

#### 2.2.5. Process of Printing

**Figure 3:**
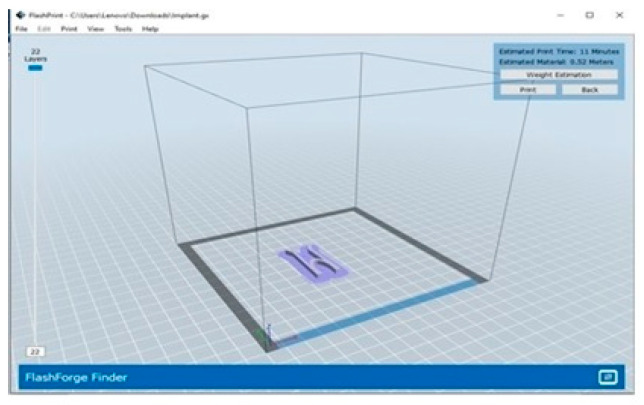
Screengrab the software FlashPrint^®^ containing the design of the stent ready to be printed.

The technique of printing using FDM (Fused Deposition Modeling). Firstly, the nozzle was set to preheat at a temperature of 220 °C, then the filament was loaded onto the nozzle of the machine. Later, the machine was connected to a laptop with the help of a USB cable. The design in the format of .STL file was sliced with the help of the software Cura^®^ and was exported to another software called FlashPrint^®^, this software is compatible with the 3D printer used. 3D Systems’ Stereolithography CAD uses the STL file format exclusively. Standard Triangle Language and Standard Tessellation Language are two of the many backronyms for STL [6]. This file format is widely utilized in the fields of rapid prototyping, 3D printing, and computer-aided manufacturing, and it is supported by a wide variety of other software applications. STL files describe the geometry of a three-dimensional object’s surfaces but not its colors, textures, or any other common attributes of CAD models. This software is where the gx code was generated, which allows printing [20]. The g-code is used in 3D printing and contains commands that allow the printer to move parts around. After providing the structure of the stent with proper support in the software, a command to print the design was given to the printer with the help of the software, which initiated the printing. Once the printing was conducted; the supports were torn off from the stent [21]. The screengrab as shown in Figure 3 shows the design of the stent, ready to be printed.

#### 2.2.6. Study of the Mechanical Properties of the Stent

##### Stress Intensity Factor

To accomplish this experiment, the structure was first imported into the software, and the material used (in this case, PLA) was selected. Then the structure was provided with fixed supports, and the force was adjusted. Then, the software helps in solving according to all the set criteria, which will result in stress, strain, and deformation. The same procedure is followed in calculating the maximum displacement [22].

##### Maximum Displacement

This test gives the relationship between force and displacement. The simulation represents the distance traveled with the application of 50 newtons. The Ansys software was used to obtain the simulated maximum displacement results for the purpose of this study. The Anysys software is an engineering simulation software developed by Anysys company.

#### 2.2.7. Evaluation of the Nasal Stent

##### Calibration Plot of MF

Prior to the drug loading onto the stent, the standard calibration plot of MF was obtained by the preparation of reference solutions with an appropriate solvent, in concentrations of approximately 10 µg/mL, 20 µg/mL, 30 µg/mL, 40 µg/mL and 50 µg/mL, that were prepared from an intermediate solution of concentration 100 µg/mL, which in turn was prepared from a first stock solution of approximately 1000 µg/mL concentration.

##### Measurement of Absorbance

The reference solutions were subjected to the measurement for absorbance in a UV-visible spectrophotometer, with the wavelength being 300 nm, which is the absorption maxima or λmax of the drug. Chloroform was used as a blank. The graph of the standard calibration was obtained by plotting the concentration of the drug on the *X*-axis and absorbance on the *Y*-axis.

#### 2.2.8. In-Vitro Biodegradation Studies

For carrying out the studies, PBS was prepared. A placebo stent without any drug loaded onto it was then kept in a 25 mL PBS solution, which was kept at a temperature of 37 °C for eight hours a day for 30 days. In pre-determined intervals, the stent was taken out, washed with Millipore water, dried in a hot air oven and re-weighed.

#### 2.2.9. Drug Loading

##### Drug Loading Was Achieved in Two Stages

Stage 1—Drug loading onto the 3D printable filaments
Stage 2—Drug loading onto the 3D-printed stent

##### Drug Loading onto the 3D Printable Filaments

The 3D printable PLA filament was cut into pieces of 1 cm and immersed in 1.5 mL of drug-containing solution, which was then incubated at a temperature of 80 °C in a water bath until the solvent was completely evaporated. After the complete evaporation of the solvent, the filaments were washed in Millipore water and air-dried for 24 h.

##### Drug Loading onto the 3D Printed Stent

**Figure 4:**
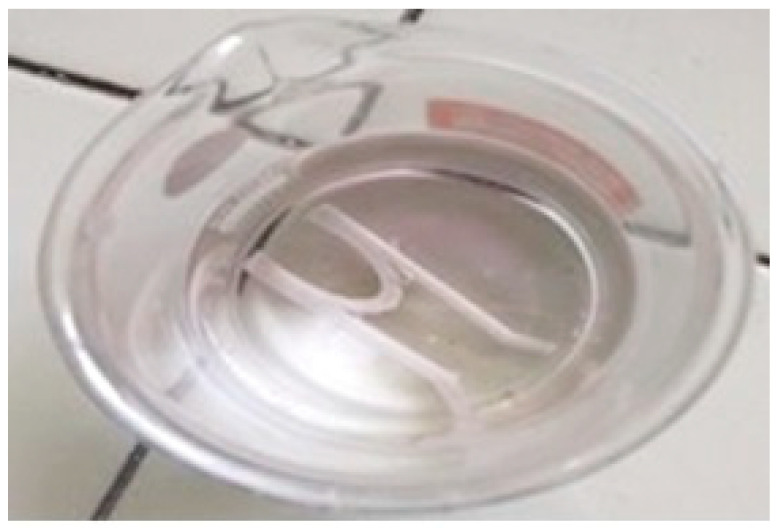
Stent in solvent-drug solution prior to incubation.

For a 3D-printed stent, the amount of solvent required for the stent’s dimensions was calculated, and a predetermined amount of drug was dissolved. In this solution, the stent was immersed. The latter processes are the same as those described in drug loading for filaments. Figure 4 shows the stent in the drug solution prior to incubation. Drug-loading of the 3D-printed stent was confirmed by three different techniques, i.e., by assessing the increase in weight of the stent after loading of the drug, by analyzing the samples with the help of UV spectrophotometry, and SEM analysis. The concentration of drug present in the stent was also determined using UV spectrophotometry. The filaments were heated to dissolve in a suitable solvent and analyzed at a wavelength of 300 nm. The obtained absorbance values were substituted in the linearity formula y= mx + c.

In the case of the stent, the shavings that were obtained from the filed stent were completely dissolved in the same appropriate solvent that was used for the filament, and the concentration of drug present was determined as mentioned above.

#### 2.2.10. In Vitro Drug Release Studies

**Figure 5:**
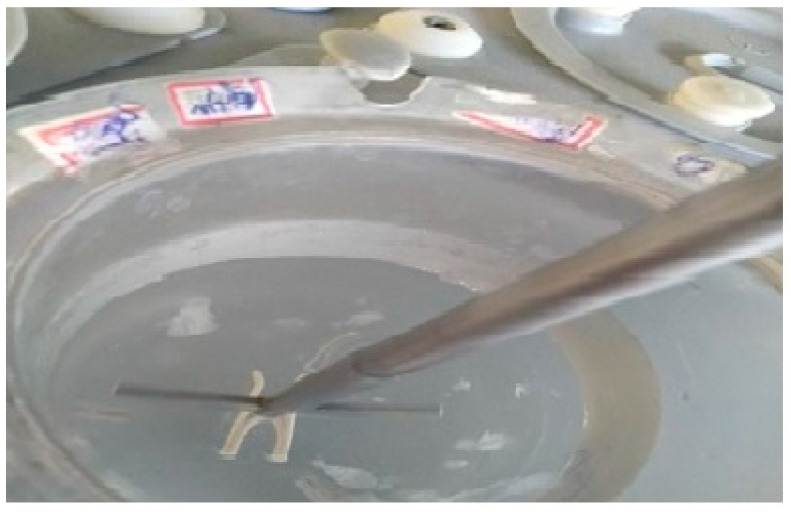
Picture of stent in the basket of the dissolution apparatus, with the paddle in action.

Release studies were conducted for a duration of eight hours; 900 mL of the 8.4 pH PBS solution was taken in a basket that was maintained at 37 ± 0.5 °C. To this, the drug-loaded stent was dropped. Figure 5 shows the setup of the dissolution apparatus. The samples were collected at 15-min intervals in the first hour, and later, the sampling was conducted every hour for the next seven hours. During sampling, a total of 10 mL of the PBS solution was pipetted out near the paddle of the arrangement in an alternating manner for each sampling to ensure consistency in sampling. To keep the sink condition, the same amount as the collected volume was replaced with fresh PBS buffer. The collected samples were analyzed for the content of MF in the UV-Spectrophotometer at the absorption maxima of 300 nm. The obtained values indicate the amount of drug released in the given sampling time slot [23,24].

The amount of drug released was calculated by the formula:

Amount of drug released = (Concentration × Dilution Factor × Volume of dissolution media)/1000(1)

%Drug release = [Amount of drug released (µg)/Dose (µg)] × 100(2)

Dilution factor is calculated by the formula

DF = Volume of the flask/Volume of the pipette(3)

## 3. Results and Discussion

**Figure 6:**
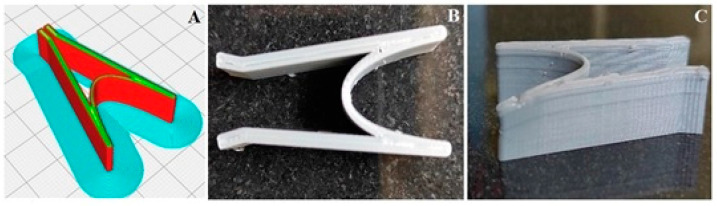
The design of the stent before (**A**) and after 3D printing (**B**,**C**).

The present research work aims to prepare a nasal stent loaded with mometasone furoate; a corticosteroid drug used in the treatment of rhinitis, asthma, and a few skin conditions. In rhinitis, it is used as an anti-inflammatory agent. PLA is the polymer used due to its biodegradability and biocompatibility, as well as because it is inexpensive. An FDM type of 3D printing is utilized for the purposes of 3D printing the nasal stent. Drug-polymer compatibility was confirmed with the help of FT-IR and DSC analysis. SEM images revealed the surface of the 3D-printed stent is free from any sharp or rough edges, and the drug-loaded stents confirmed the presence of the drug in the form of scattered white specks throughout the field of observation. Drug loading on the stent is conducted by the solvent casting method; with an average of 72.8% drug being loaded onto the stent. The released drug from the stent was studied with the help of the dissolution apparatus, and it was concluded that the stent shows good, constant release characteristics. Biodegradation studies were carried out for 30 days, where a good amount of degradation was achieved. Various prototypes of the intended design were created, and finally, the design with the hairpin-like mechanism was finalized due to its simple design, simple mechanism, and ease of 3D printing. The physician assists in stenting the device after endoscopic sinus surgery. Its spring-like design props open when deployed from a silicone catheter. The spacing function provided by the stent along with the loaded drug, not only assists in wound healing and decreases inflammation, but also minimizes scar tissue and granulation tissue formation over a period of 30 days before its complete disintegration. Figure 6 represents the design of the stent before (Figure 6A) and after 3D printing (Figure 6B,C). The height, width, and depth of the sinuses not only show a considerable ethnic difference, but sexual dimorphism also plays an essential role. Both of these factors contribute significantly to the differences. The mechanical dimensions of the finalized design were within the mean values of length (in males, 33.4 ± 4.6 mm, and in females, 30.9 ± 4.2 mm); the mean value of width was, in males, 25.4 ± 4 mm and in females, 23.3 ± 3.9 mm. API Characterization of Mometasone Furoate (IP) [as per the analysis report provided by Coral Drugs Private Limited]. The analysis report for MF is compiled in Table 2. The drug showed optimum results for λmax at 300 nm. The drug was subjected to solubility studies using some solvents (saturation solubility), and the solubility can be described in the following order: water < methanol, ethanol, and isopropyl alcohol (slightly soluble) < Acetone and chloroform (soluble) < Tetrahydrofuran (freely soluble) [25].

- Optimization of formulation, Nasal stent design
- The optimization of the formulation in terms of design;
- The design was one of the biggest aspects of the research. The design was to be such that the stent opens up by itself when deployed in the maxillary sinus.
- The design facilitates the prevention of closing up of the sinus again after surgery due to inflammation.
- The design was to be compliant with 3D Printing.
- Rough, sharp edges were to be avoided in the design to not cause further damage to the nasal mucosa.
- Once the design was finalized, the appropriate opening mechanism of the same was checked.

**Figure 7:**
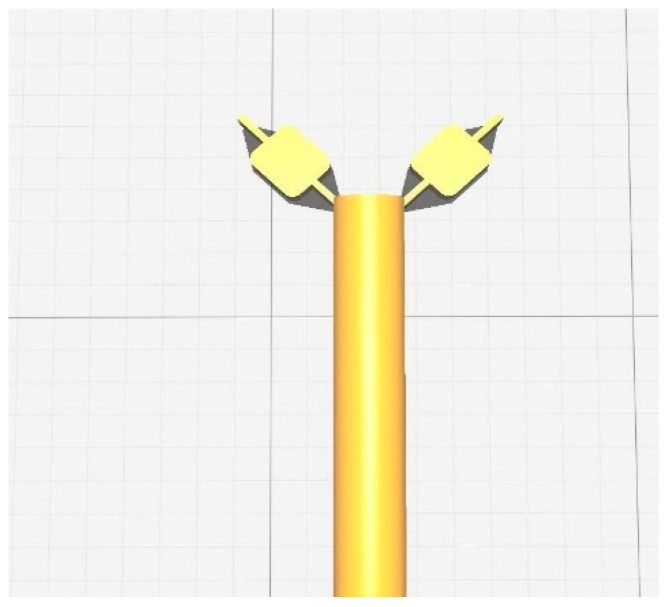
A screengrab of the process of designing the implant in .STL format.

The optimized design of the stent is used for the formulation. A screengrab of the process of designing the implant in .STL format is represented in the Figure 7.

### 3.1. FT-IR Spectroscopy Analysis

**Figure 8:**
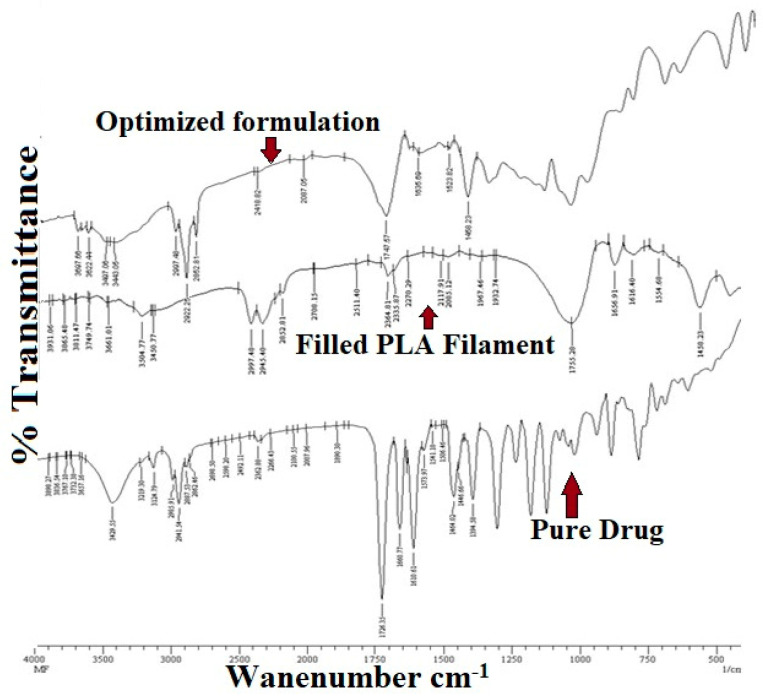
FT-IR Spectra of optimized formulation, filled PLA filament and pure drug.

Pure drug and formulation of drug and polymer sample were subjected to the analysis. The spectral readings of the pure drug showed characteristic peaks, which were subjected to comparison with the characteristic peaks obtained from the spectral readings of the drug-polymer mixture. The lack of a significant change in the characteristic peaks in both spectra indicates the absence of drug and polymer interactions between them. Table 3 shows the interpretation of FT-IR data. The FT-IR spectra of optimized formulation-filled PLA filament and pure drug are depicted in Figure 8.

### 3.2. Differential Scanning Calorimetry (DSC)

**Figure 9:**
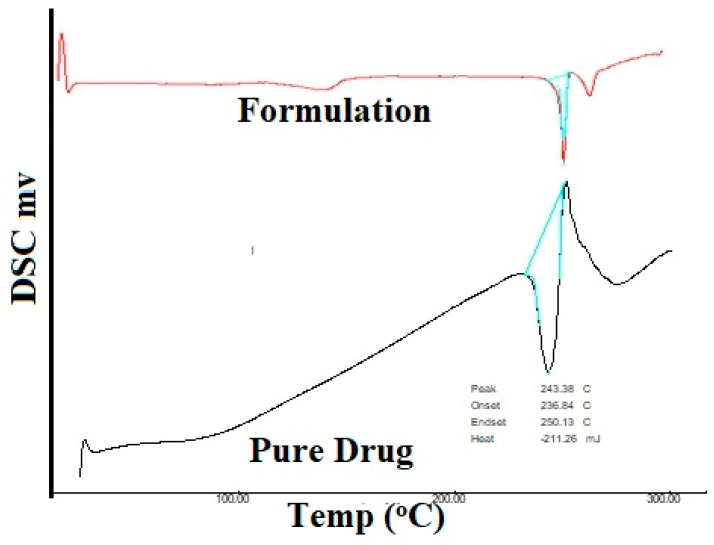
DSC Thermogram of the pure drug and formulation.

DSC thermograms of a pure drug sample and an optimized formulation sample are represented in Figure 9. The DSC curve of MF shows two events. One endothermic event at 243.38 °C which is consistent with the prior literature, and an exothermic event at approximately 260 °C. The DSC curve of the optimized formulation also shows one endothermic event at 249.01 °C and an exothermic event at approximately 260 °C. The energy consumed by pure MF is −211.26 mJ and the energy consumed by the formulation is −187.51 mJ. With the energy consumed by both samples being almost similar and the endothermic events on both pure drug and the final formulation being very similar, it can be concluded that the polymer and the drug show good compatibility.

### 3.3. SEM Analysis

**Figure 10:**
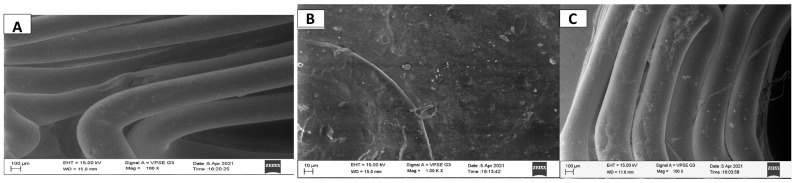
SEM image of the stent without drug, drug-loaded stent taken at different angles and at different magnifications.

SEM was used to study the surface morphology of one stent, as well as for the confirmation of drug loading. There is a visible difference between the stent without the drug; (shown in Figure 10A) and the stent with the drug (shown in Figure 10B,C). In the stent without drug, the surface is free of any cracks and drug particles are absent throughout the field of observation. In the stent with the drug also, the surface is free of any cracks and dents; however, the drug particles are clearly visible as white specks scattered throughout the field of observation.

### 3.4. Mechanical Properties of the Nasal Stent

**Figure 11:**
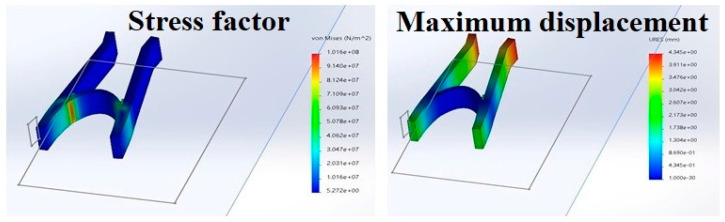
Mechanical properties of the nasal stent showing stress factor and maximum displacement.

In the picture, the red highlighted part shows the part of the geometry that is under maximum stress and strain when an external force is applied. This signifies that the part under too much stress is prone to deformation, which may lead to bending and snapping. As apparent from Figure 11, the part highly susceptible to too much stress has very little surface area; meaning the structure is not at a very high risk of snapping and breaking when put under stress [11]. From Figure 11, when 50 newtons are applied; the maximum displacement of the ends of the structure causes a deformation of 1.03993. This again signifies that the distance traveled by the ends of the structure will not cause any significant harm to the integrity of the structure under stress.

### 3.5. Evaluation of the Drug Loaded 3D Printed Nasal Stent

#### 3.5.1. Drug Loading

**Figure 12:**
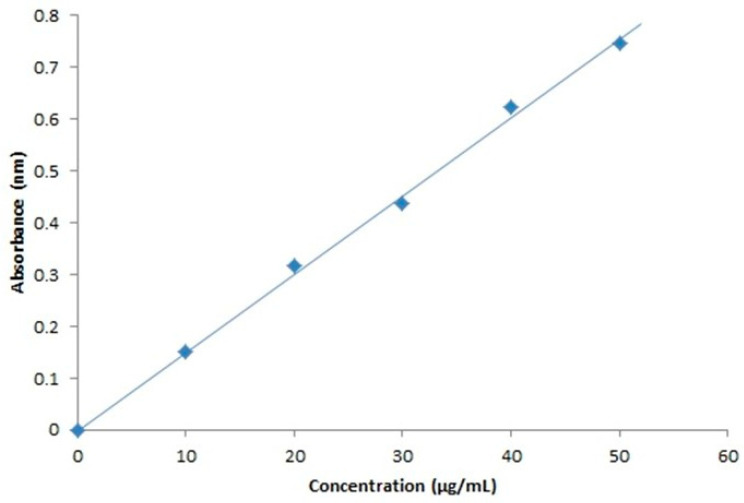
Standard calibration curve of mometasone furoate at 300 nm.

The standard calibration curve indicates that the drug obeys Beer’s law. The optimized result could be found around 10–50 µg/mL. The concentration of the drug was calculated using the linear regression equation (R^2^ = 0.996) as shown in Figure 12.

#### 3.5.2. Drug Loading to 3D Printable Filaments

**Figure 13:**
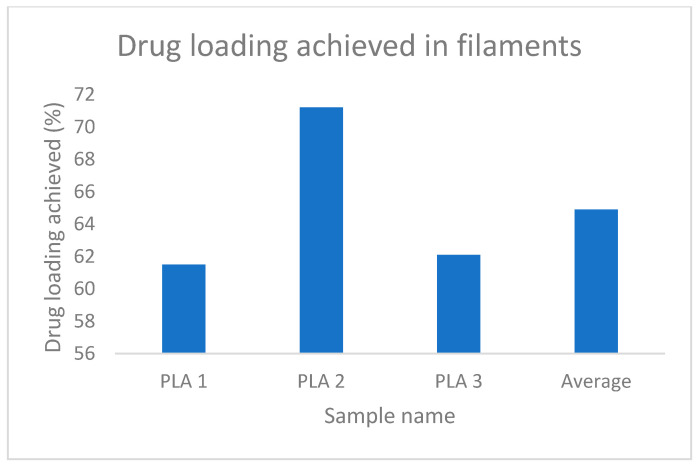
% drug loading achieved in filaments.

**Figure 14:**
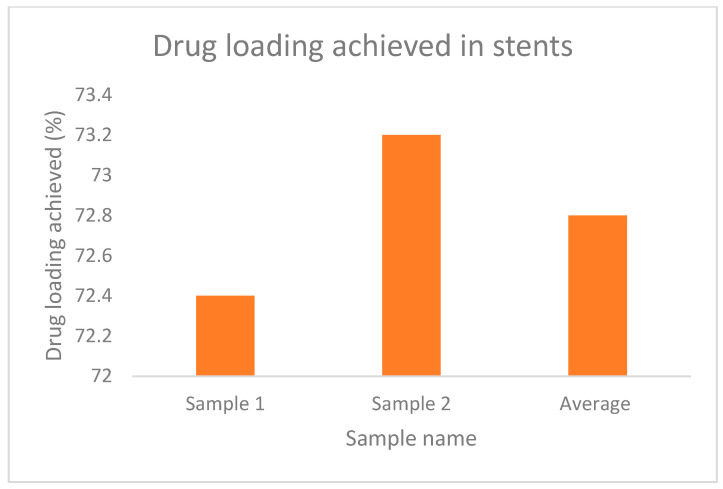
% drug loading achieved in stents.

**Figure 15:**
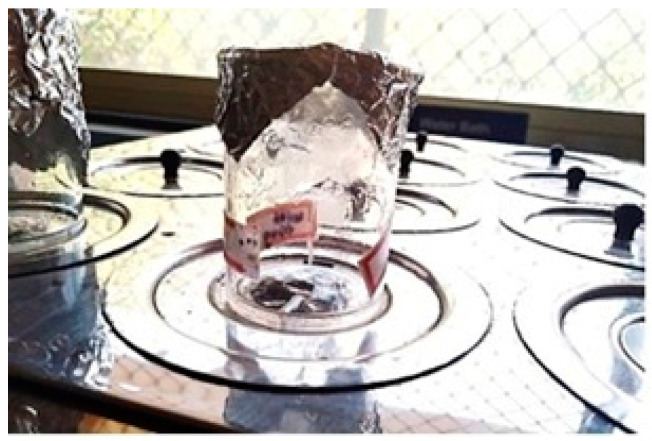
Filaments on hot water bath.

The increased weight of the filaments and stent, which indicates the loading of the drug is evident from Table 4 and Table 5, whereas, Figure 13 and Figure 14 represent the percentage of drug loading achieved by the stent and filament, respectively. Figure 15 represents filaments in a hot water bath.

### 3.6. In-Vitro Biodegradation Studies

**Figure 16:**
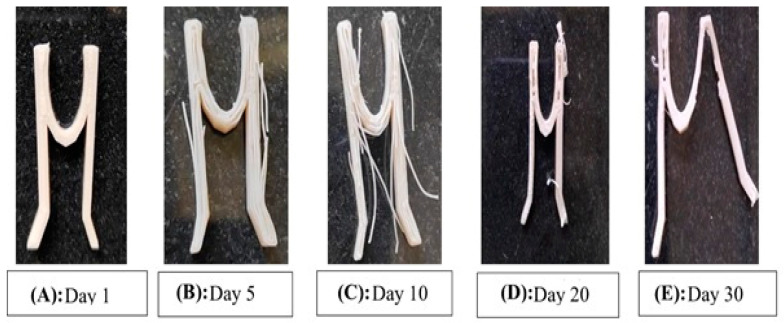
The progression of the degradation of the stent.

**Figure 17:**
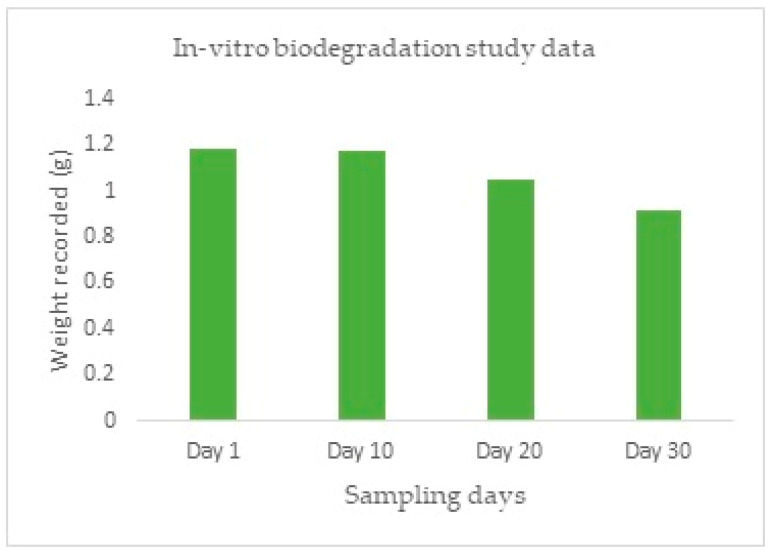
In-Vitro biodegradation study.

The progression of the degradation of the stent is represented in Figure 16 after it was subjected to an increased rate of degradation at different periods. From Figure 16A–E, it is possible to predict the degradation pattern of the structure. Figure 17 shows the decreasing weight of the stent during the pre-determined testing intervals.The degradation can be proved by the decreased weight of the structure, as well as the visibly decreased integrity in the structure [24].

### 3.7. In Vitro Drug Release Profile

**Figure 18:**
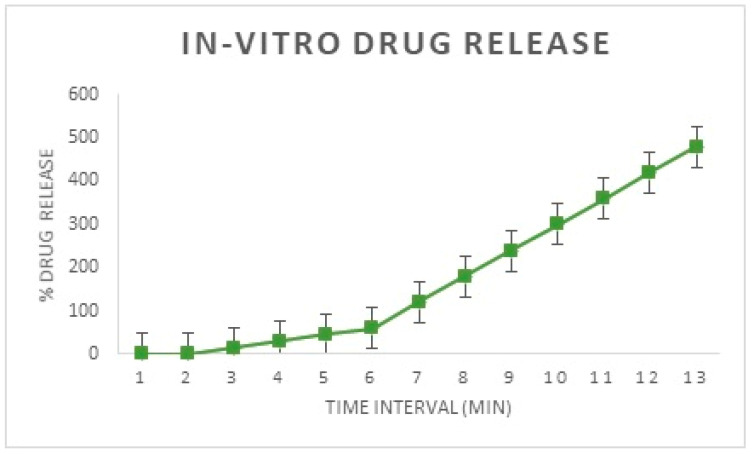
In-Vitro drug release from the developed implant.

From Figure 18 it is evident that the amount of drug released is negligible in the first eight hours, as desirable for this study. However, from the first hour, a steady increase in drug release is observed and the drug release is at an almost constant rate at the seventh and eighth hours. This shows that a constant rate of drug release is possible from the finalized design of the stent, which nullifies the need for coating with a second polymeric solution [26].

## 4. Conclusions

The current project aims to develop a nasal stent loaded with the drug MF, which possesses the following advantages. A drug-loaded stent reduces post-surgical inflammation and accelerates the healing process. The design of the stent provides a spacing function that prevents the sinus from closing again due to inflammation and scar tissue formation, which also necessitates the healing process. The stent should be economical and easily accessible to the Indian population. The stent should be biodegradable; so that a secondary intervention is not required to take it out after its intended period of residence in the sinus. PLA polymer is readily available, inexpensive, biocompatible, and biodegradable; hence, it finds a great number of applications in the medical field. From the evidence of prior literature, it was found that the drug loading onto this polymer filament is possible with impressive results. For these reasons, the PLA was finalized. The drug was successfully loaded onto the stent with 72.8% of the drug being successfully loaded onto the stent. The release of drug from the stent was studied; with the drug release being constant and in a desirable manner. Biodegradation of the stent treated with PBS was carried out for 30 days; where a good amount of the stent degraded under a specific set of pre-set simulated conditions based on the in-vivo conditions. The design of the stent is finalized and is similar to a hairpin-like mechanism.

## 5. Future Perspectives

The field of biodegradable nasal stent research offers significant opportunities for innovation and advancement. Nasal stents are medical devices used to support the nasal airway and prevent its collapse. Biodegradable nasal stents are designed to gradually degrade over time and eventually dissolve within the body, eliminating the need for removal surgery. The use of biodegradable materials in nasal stents offers many advantages, including reduced risks of infection and inflammation, improved patient comfort, and lower healthcare costs.

Drugs such as hyaluronic acid can be incorporated along with mometasone furoate. HA is known to be compatible with MF, and it can be used as a plasticizer to increase the penetration of MF into the nasal mucosa. HA acid is extensively studied for its wound-healing properties, which could also help in accelerating the wound-healing process post-surgery by preventing the complications such as synechia and stenosis. A formulation is available in the market with the combination of MF-HA in the form of a cream, meant for topical application. Another drug that could be investigated for the same purpose is N-acetyl cysteine. The author recommends a longer duration of dissolution studies for at least 24 h to one month to obtain a clearer release pattern of the drug. After longer dissolution studies, if needed, a thin coating of the stent can be conducted with either PCL, chitin or chitosan. The coating could help in making the stent exhibit sustained release characteristics. The author also recommends longer biodegradation studies for the stent, for at least three months until a complete degradation is achieved.

## Figures and Tables

**Table 1:** Overview of treatments and available dosage forms for rhinosinusitis.

Sl. no	Treatments	Dosage Forms	Approach Type
1	Rinsing the nasal passages several times a day	Saline nasal spray	Traditional
2	Nasal Corticosteroids to help prevent and treat inflammation	Nasal Sprays	Traditional
3	Nasal decongestants	Over-the-counter (OTC) prescription tablets nasal sprays and liquids,	Traditional
4	Pain relievers	OTC drugs such as acetaminophen, ibuprofen (Tablets, syrups and others)	Traditional
5	Antibiotics	Oral, topical dosage forms.	Traditional
6	Immunotherapy	Injection	Traditional
7	Packing materials	Hydrogel, gels.	Novel
8	Injectable space-filling gels	Gels	Novel
9	Nasal stent	Made up of biodegradable, biocompatible polymers.	Novel

**Table 2:** API Characterization of MF.

Sl. no	Test	Specification	Result
1	Description	A white or almost white powder	White powder
2	Solubility	“Insoluble in water, soluble in acetone and methylene chloride, slightly soluble in 95% ethanol”	Complies
3	Identification by IR	The IR spectrum of the sample should be concordant with that of a similar preparation of standard	IR spectrum of the sample is concordant with that of a similar preparation of standard
4	Sulfated ash	Not more than 0.1%	0.03%
5	Specific Optical Rotation	+50° to +55°	+53.09°
6	Loss on drying	Not more than 0.5%	0.28%
7	Related Substances (by HPLC) Any single impurity Total impurites	Not more than 0.3% Not more than 0.6%	0.18 0.18
8	Assay (by UV)	97.0% to 102.0% (on dried basis)	100.34%
9	Heavy metals	Not more than 30 ppm	Less than 30 ppm
	Residual solvents (By HSGC) Methanol Acetone Methylene chloride Benzene	Not more than 3000 ppm Not more than 5000 ppm Not more than 600 ppm Not more than 2 ppm	23 ppm 889 ppm 7 ppm not detected
10	Particle size (by Malvern Masterizer)	D(0.9) should be less than 10.0 µm	4.01 µm

**Table 3:** IR interpretation and comparison between MF and PLA.

Sl. no	Characteristic Peaks Observed	Functional Group
1	3497.06 cm^−1^	−OH alcohols
2	1750, 1700, 1640, cm^−1^	>C=O (3)
3	1300 cm^−1^	C−O

**Table 4:** Weight variation of filaments-comparison of before and after drug loading.

Sample Name	Weight of the Filament before Drug Loading (g)	Mean ± SD (n = 3)	Weight of the Filament after Drug Loading (g)	Mean ± SD (n = 3)
PLA1	0.028		0.032	
PLA2	0.030	0.029 ± 0.0006	0.036	0.034 ± 0.0008
PLA3	0.029		0.033	

**Table 5:** Weight variation of stents-comparison of before and after drug loading.

Sample Name	Weight of the Filament before Drug Loading (g)	Mean ± SD (n = 2)	Weight of the Filament after Drug Loading (g)	Mean ± SD (n = 2)
Sample 1	1.186	1.186 ± 0.00410	1.191	1.191 ± 0.00526
Sample 2	1.186		1.190	

## Data Availability

The authors confirm that the data supporting the findings of this study are available within the article.
